# How can a joint European health technology assessment provide an ‘additional benefit’ over the current standard of national assessments? Insights generated from a multi-stakeholder survey in hematology/oncology

**DOI:** 10.1186/s13561-022-00379-7

**Published:** 2022-06-02

**Authors:** Elaine Julian, Fabrizio Gianfrate, Oriol Sola-Morales, Peter Mol, Jean-François Bergmann, Tomas Salmonson, Ansgar Hebborn, Mathilde Grande, Jörg Ruof

**Affiliations:** 1 r-connect ltd, Basel, Switzerland; 2 University of Ferrara, Ferrara, Italy; 3 Fundació HiTT, Barcelona, Spain; 4 Department of Clinical Pharmacy and Pharmacology, University Medical Center Groningen, University of Groningen, Groningen, The Netherlands; 5 University Paris-Cité and AP-HP, Paris, France; 6 Consilium, Uppsala, Sweden; 7 efpia, Brussels, Belgium; 8 AMEDICONSEIL, Brive-la-Gaillarde, France; 9 Medical School of Hanover, Hanover, Germany

**Keywords:** EU HTA, Europe’s Beating Cancer Plan, Comparators, Endpoints, Clinical trial design, Real world evidence, Patient-relevance, Access

## Abstract

**Objectives:**

We conducted a multi-stakeholder survey to determine key areas where a joint European health technology assessment (HTA) could provide ‘additional benefit’ compared to the status quo of many parallel independent national and subnational assessments.

**Methods:**

Leveraging three iterative Delphi cycles, a semiquantitative questionnaire was developed covering evidence challenges and heterogeneity of value drivers within HTAs across Europe with a focus on hematology/oncology. The questionnaire consisted of five sections: i) background information; ii) value drivers in HTA assessments today; iii) evolving evidence challenges; iv) heterogeneity of value drivers across Europe; v) impact of Europe’s Beating Cancer Plan (EBCP). The questionnaire was circulated across *n* = 189 stakeholder institutions comprising HTA and regulatory bodies, clinical oncology associations, patient representatives, and industry associations.

**Results:**

*N* = 30 responses were received (HTA bodies: 9; regulators: 10; patients’ and physicians’ associations: 3 each; industry: 5). Overall, 17 countries and EU level institutions were represented in the responses. Consistency across countries and stakeholder groups was high. Most relevant value drivers in HTAs today (scale 1, low to 5, high) were clinical trial design (mean 4.45), right endpoints (mean 4.40), and size of comparative effect (mean 4.33). Small patient numbers (mean 4.28) and innovative study designs (mean 4.1) were considered the most relevant evolving evidence challenges. Heterogeneity between regulatory and HTA evidence requirements and heterogeneity of the various national treatment standards and national HTA evidence requirements was high. All clinical and patient participants stated to have been with EBCP initiatives.

**Conclusions:**

For a European HTA to provide an ‘additional benefit’ over the multitude of existing national assessments key methodological and process challenges need to be addressed. These include approaches to address uncertainty in clinical development; comparator choice; consistency in approaching patient-relevant endpoints; and a transparent and consistent management of both HTA and regulatory procedures as well as their interface, including all involved stakeholder groups.

**Supplementary Information:**

The online version contains supplementary material available at 10.1186/s13561-022-00379-7.

## Introduction

Health Technology Assessments (HTA) have been introduced in almost all European Union Member States. However, EUnetHTA’s (European Network for Health Technology Assessment) 2018 report [1] as well as a recently published comparison of EUnetHTA’s Relative Effectiveness Assessments and the respective national procedures in key European markets [2] revealed that HTA working practices and appraisals differ considerably across Europe. Availability and content of methodological guidance documents as well as national reimbursement processes show large differences within the various European countries [3–5] with the related ‘funding ecosystems’ being in constant transition [6].

In December 2021 the EU HTA Regulation, a key pillar of the EU Pharmaceutical Strategy, was adopted by the Council and the European Parliament. It aims to harmonize methodological standards and to foster collaboration among European HTA bodies. The regulation came into force in January 2022, formally representing the start of the implementation work, and will be applied from January 2025 [7, 8]. The Council’s bridging activities until 2025 include a tender agreement with the newly formed EUnetHTA21 consortium, allowing a very limited number of joint clinical assessments and joint scientific consultations, as well as further evolving and strengthening the methodological basis for European HTAs [9, 10].

The European HTA regulation will be adopted in a stepwise approach. From 2025 onwards all new cancer medicines and advanced therapy medicinal products (ATMPs) will be jointly assessed ( [8], article 7.2) while the final HTA appraisals and the subsequent reimbursement decisions remain within the remit of each member state ( [8], Preamble §14). From 2028 joint clinical assessments will also apply to orphan medicinal products. The initial focus and statements of the Councis’s representatives [7] suggests an implicit link of the HTA regulation to Europe’s Beating Cancer Plan (EBCP), a main priority of the current European Commission in the area of health and a key pillar of a strong European Health Union [11]. Therefore, the implementation of the HTA regulation targets the most innovative and challenging medicines in terms of assessment and comparative clinical trial design upfront.

We conducted a multi-stakeholder survey to determine key areas where a Joint European HTA could provide ‘additional benefit’ compared to the status quo of many parallel independent national and subnational assessments.

## Methods

### Development of the questionnaire

Leveraging three iterative Delphi cycles [12], a questionnaire was developed covering evidence challenges and heterogeneity of value drivers within HTAs across Europe with a focus on hematology/oncology, and the EBCP. Experts included in the panel covered a wide variety of European national (France, Germany, Italy, Spain, Sweden) and institutional backgrounds (Clinical, Academic, Regulatory, HTA body, Payer, Industry).

The questionnaire is included as supplementary material. The iterative Delphi methodology was modified and did not include formal ranking and scoring of the panel’s responses. Instead, two of the authors (JR/ EJ) proposed an initial item pool that was subsequently commented on by the other authors. After refinement of the questions a second round of review was conducted. Finally, the electronic version of the questionnaires was developed. All other authors were asked to fill in the questionnaire and provide final comments and recommendations. Both qualitative and quantitative questions were included.

Stepwise, through structured web-based video meetings, convergence was reached within the Delphi panel. Key adjustments throughout the development of the questionnaire included:

- First round of review:

*Key discussion points within the Delphi panel included the structure of the questionnaire and the format of the responses. Thus, the initial item pool was structured into five different questionnaire sections. Furthermore, the response type of many questions was adjusted to a semi-quantitative format to allow for ranking as well as for many free-text responses: i) background of the respective institution in relation to the market access process (qualitative questions and dichotomous yes/no questions); ii) key value drivers within the current benefit assessments of oncology medicines (Likert response items from 1 [low relevance] to 5 [high relevance] as well as qualitative free text questions); iii) evolving evidence challenges in oncology medicine assessments (Likert response scales from 1 [low relevance] to 5 [high relevance] as well as qualitative free text responses); iv) heterogeneity of value drivers across Europe (qualitative questions); v) the impact of the EBCP on the respondent’s institution (qualitative as well as quantitative questions).*

- Second round of review:

*While the revised version structure of the questionnaire was consented within the panel, the level of detail of some of the questions was discussed controversially. A decision was reached to separate a more detailed version aimed for HTA bodies and a shorter version for any other stakeholder group.*

- Third round of review:

*Within the third meeting of the panel feasibility and implementation challenges were addressed. An introductory section was added to the questionnaire providing some background and the scope of the survey as well as information on data privacy and the handling of any responses received. Furthermore, some questions were shortened to reach an overall response time of ~ 15 minutes.*

### Circulation of the questionnaire

Subsequently, the questionnaire was circulated across stakeholders that were considered to be deeply involved in the implementation of the HTA regulation i.e., i) European and national HTA bodies; ii) European and national regulatory bodies; iii) European and national clinical hematology/ oncology associations; iv) European and national patient representatives (focus on hematology/ oncology); and v) industry associations both on an EU and national level. To develop the distribution list we utilized both public sources as well as the insights and the network of all Delphi panel participants. Specifically, the target recipients of the questionnaire included:

- HTA bodies: EUnetHTA and all HTA bodies participating in the former EUnetHTA/current EUnetHTA21 network,
- Regulatory bodies: The European Medicines Agency (EMA) and all national regulatory agencies listed on the European Medicines Agency website,
- Hematology/oncology societies: The European Society of Medical Oncology (ESMO) as well as the European Cancer Organisation and all of ESMO’s national member organisations,
- Patient organisations: The European Patients’ Forum, the International Alliance of Patients Organizations (IAPO), the European Cancer Patient Coalition (ECPC), and the Association of European Cancer Leagues (ECL), as well as patient associations for specific oncological disorders and key national patient associations,
- Industry: The European Federation of Pharmaceutical Industries (efpia), national pharma trade associations as well as individual pharmaceutical manufacturers.

While Turkey is not an EU member state, the Turkish Trade Association AIFD is a member association of efpia and the Turkish Society of Medical Oncology is a member of ESMO. Therefore, those two organisations were included in the distribution list for the questionnaire. Contact details of those stakeholder institutions were identified. Subject to availability distinct individuals within any of those institutions were contacted by mail. For all institutions without the availability of personal contacts the official e-mail contacts were used.

The questionnaire was shared with all identified stakeholders simultaneously. Ten days later a reminder e-mail was circulated. A second reminder was circulated after 3 weeks.

### Data handling and analysis of the questionnaire

All questionnaire responses received were pseudonymized prior to any analysis. Data were stored on a password-protected separate file. Data were transferred into a predefined Excel file. Double-entry technology (JR/ EJ) was applied. Any qualitative comments received were included into the Excel file. Prespecified descriptive analyses were conducted on the quantitative items of the questionnaire. Exploratory post-hoc analyses were performed linking the outcomes to the key work packages of EUnetHTA21.

## Results

**Fig. 1:**
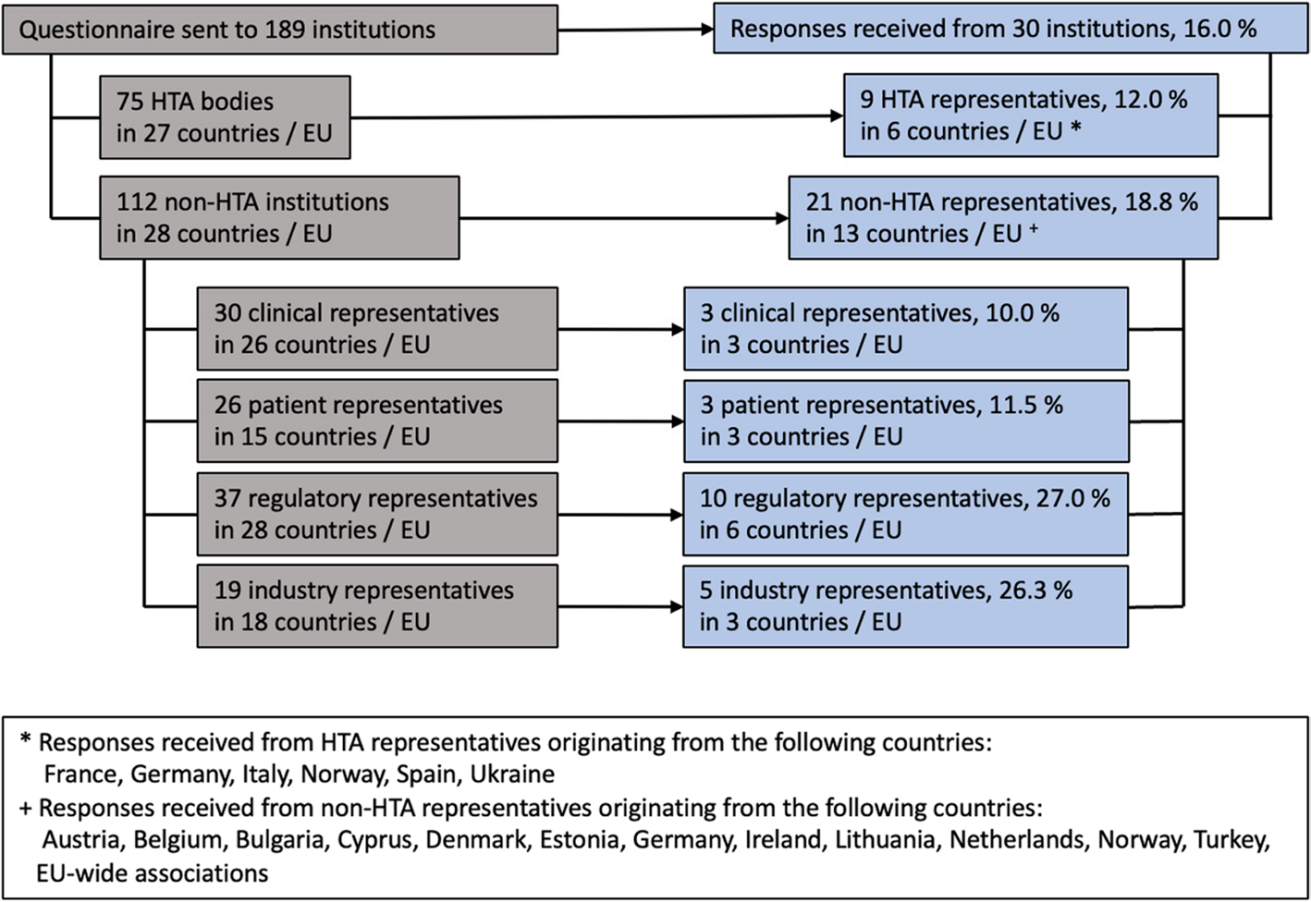
Questionnaire distribution and responses received

The online survey was shared with a total of 189 stakeholder institutions (Fig. 1). *N* = 30 responses were received (HTA bodies: 9; regulators: 10; patients’ and physicians’ associations: 3 each; industry: 5). Overall, 17 national and EU level representatives (European Union institutions and Austria, Belgium, Bulgaria, Cyprus, Denmark, Estonia, France, Germany, Ireland, Italy, Lithuania, Netherlands, Norway, Spain, Turkey, Ukraine) are represented in the responses.

### Background of the respective institutions

Among the 21 ‘other stakeholders’ respondents (i.e., regulators, clinicians, patients, industry) 12 confirmed prior experience and involvement with HTA, seven have no or low involvement with HTA, and two did not provide respective comments.

Among the nine HTA bodies 56% (*n* = 5) conduct both assessments and appraisals, while 44% (*n* = 4) only cover appraisals. Methodological guidances are published by 67% (*n* = 6) of the responding HTA bodies. The ESMO Magnitude of Clinical Benefit Scale ratings are considered by 56% (*n* = 5) of the responding HTA bodies. A total of 33% (*n* = 3) of the HTA bodies are involved in the assessment of cancer diagnostics. Registry data are taken into account by 44% of the (*n* = 4) of the HTA bodies. Past EUnetHTA assessments were only considered by two of the responding HTA bodies.

### Key value drivers within the current benefit assessments of oncology medicines

**Fig. 2:**
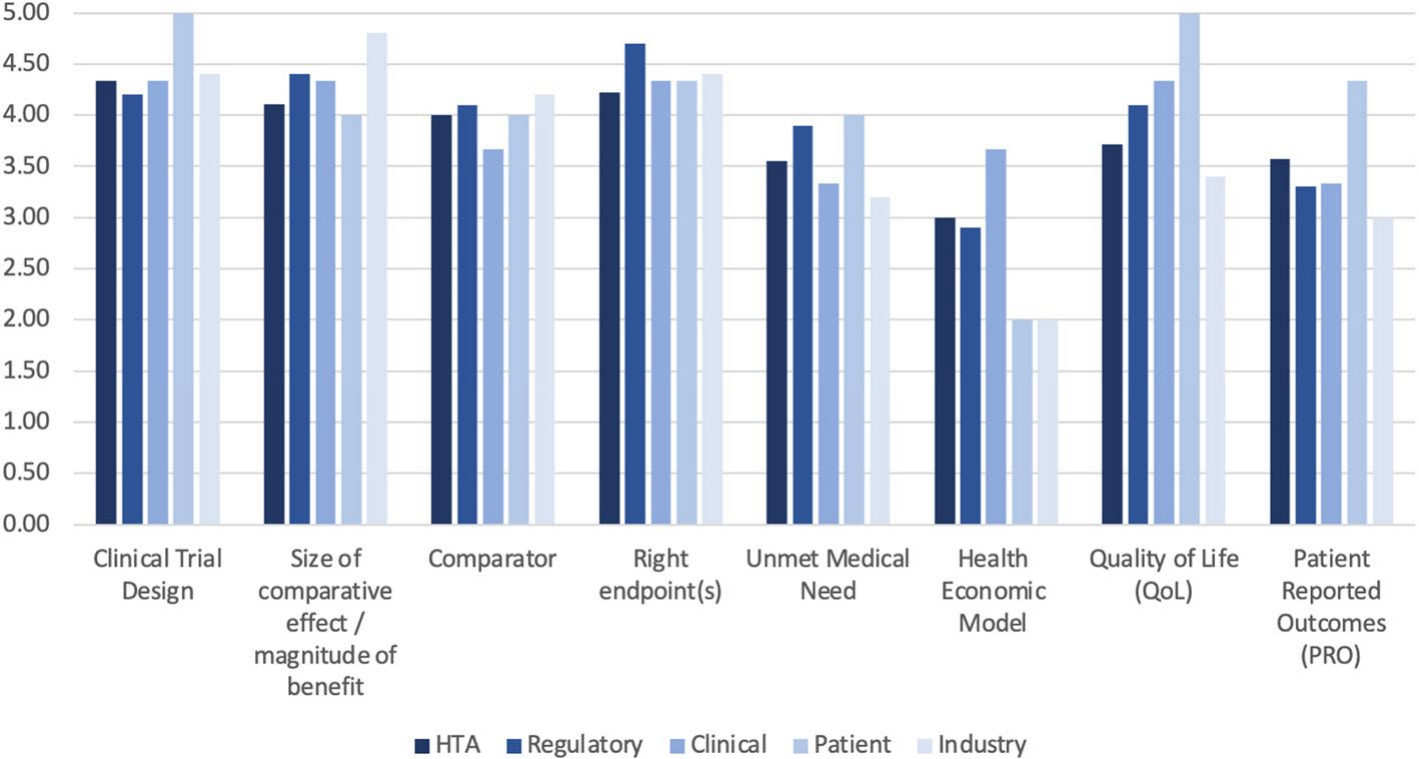
Most relevant value drivers for positive health technology assessments in oncology medicines today (Mean per stakeholder group)

An overview of the current value drivers in HTAs of oncology medicines is displayed in Fig. 2. The scale ranged from 1 (low relevance) to 5 (high relevance). The mean was highest for the items: clinical trial design (4.45), right endpoints (4.40), size of comparative effect (4.33), 4.11 for quality of life, 3.99 for comparator, 3.60 for unmet medical need, 3.51 for patient-reported outcomes, and lowest (2.71) for health economic model. Consistency across the various stakeholder groups was high with patients giving the maximum score of 5 to clinical trial design and quality of life, industry putting most emphasis (score 4.80) on the size of the comparative effect/magnitude of benefit, and both industry and patients scoring low (score 2.0) on health economic model.

### Evolving evidence challenges in oncology HTAs

**Fig. 3:**
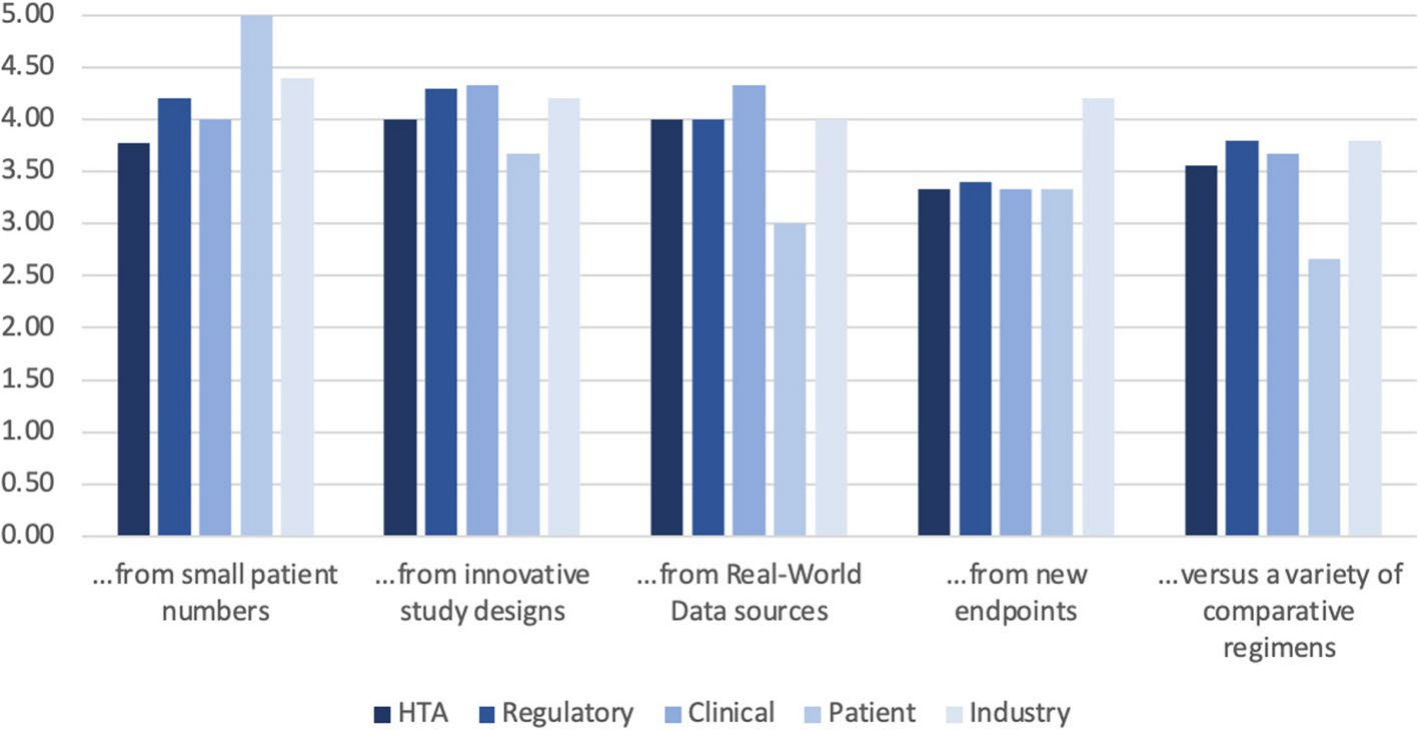
Evolving challenges for development of comparative evidence in oncology medicines (Mean per stakeholder group). The questions that was asked in the questionnaire: *‘A key challenge for future oncology medicines is deriving comparative evidence..’*

An overview of the rating of key challenges for deriving comparative evidence for oncology medicines is displayed in Fig. 3. Again, the Likert response scale from 1 (low relevance) to 5 (high relevance) was applied. The mean was highest for the challenges posed by: ‘small patient numbers’ (4.28) and ‘innovative study designs’ (4.1), 3.87 for ‘real-world data sources’, 3.52 for ‘new endpoints’ and lowest (3.5) for a ‘variety of comparative regimens’. Consistency across the various stakeholder groups was high with patients giving the maximum score of 5 to the challenge of ‘small patient numbers’, a score of 3 to the challenge posed by ‘real-world data sources’, and a score of 2.67 to the challenge of handling a ‘variety of comparative regimens’.

Free-text responses were received within this section of the questionnaire. Those responses were grouped into four categories: i) managing uncertainty; ii) comparator choice; iii) endpoints; and iv) process challenges. Results are provided in Table 1. A key element that was raised repeatedly is that methodological standards should match the shifting treatment paradigm in hematology/oncology towards ever more targeted treatments for smaller patient populations.

**Table 1:** Evolving evidence challenges for a European health technology assessment in oncology as derived from the qualitative comments provided in the questionnaire

Management of Uncertainty	Comparator Choice	Endpoint Selection	Process Challenges
*How can EU HTA take into account evolving clinical research and development paradigms in oncology to be a driver not a bottleneck for patient access to new medicines while at the same time not reducing quality standards?* *How to approach a gradually evolving evidence body with limited certainty at time of launch?* *How can certainty with small numbers* e.g.*, in childhood cancer or in ATMPs be achieved?* *Is there a case for context-related evidence requirements & standards?* *What are suitable and achievable comparative evidence requirements for real-world data and new clinical trial designs?* *How relevant are the results of the pre-defined testing hierarchy (Alpha Risk Spending) to HTAs?* *How can combination of drugs be approached in HTA in terms of benefit per drug / shared benefit,* etc.*?*	*How to manage an ever more diverging and fast-moving comparator landscape in targeted oncology?* *How to ensure that comparator choice fulfils both, regulatory and HTA requirements?* *How to manage different national standards of care and heterogeneous guideline recommendations?* *Who should be involved in the selection of appropriate comparative therapy?*	*How to ensure that endpoints fulfil both, regulatory and HTA requirements?* *How to develop and validate innovative / new endpoints that best demonstrate the benefit of a new oncology medicine to patients?* *How to include patients and clinicians in the definition and selection of endpoints that are both, relevant for clinical trials and clinical practice?* *How to determine and develop valid and meaningful threshold for minimal clinical important difference?* *How to harmonize hierarchy of endpoints and measurement methods across regulatory and HTA bodies?* *How to align the EU HTA bodies on key oncology endpoints such as PFS?*	*How can regulatory and HTA requirements be coordinated and aligned?* *How to approach the challenge of multiple PICOs?* *How to achieve harmonization in implementation of EU HTAs while the assessments are not binding for national bodies?* *How can equal access of patients to new oncology medicines be facilitated despite the joint assessments not being binding?* *How to improve inclusion of eastern European countries in this process?* *How to quantify/ track the reduction of administrative burden?* *How do evolving regulatory pathways match HTA processes* e.g.*, the upcoming new EMA regulation on orphan designation?* *How can gathering of HTA input very early in a medicine’s lifecycle with continuous follow up be facilitated?* *What if there are not sufficient slots available for early consultations?* *How can involvement of clinician and patient experts / integration of their views on key questions be improved?* *How will transparency of processes and decisions be implemented and tracked?* *How can divergence between countries in terms of expectations for EU HTA adoption (*e.g.*, depending on national reimbursement system, use of HTA for patient access* vs. *for price negotiations, national economic models,* etc.*) be minimized?* *How can it be ensured that patient access to innovation is not hindered despite the divergence in expectation by different countries?*

### Heterogeneity of value drivers across Europe

This section included only free-text items. Respondents were asked to determine the methodological heterogeneity across Europe that they would consider most (or second most, respectively) challenging and provide the rationale for their choice. A multitude of detailed comments were provided covering a variety of procedural and methodological challenges. Consistency across countries and stakeholder groups was high. Results are summarized in Table 2. The key areas that were repeatedly mentioned included heterogeneity:

- of European regulatory and applicable national HTA evidence requirements,
- of national standards of care, treatment algorithms, and guideline recommendations,
- of methodological standards for national HTAs, e.g., with regards to endpoints, comparators, or acceptance of indirect treatment comparisons, and
- of national HTA and reimbursement processes and timelines across Europe

**Table 2:** Heterogeneity of value drivers across Europe; comments received within the questionnaire

Area of Heterogeneity	Examples
Different evidence requirements for European regulatory vs applicable national HTA procedures	*• Primary study endpoints to be acceptable for regulatory authorities and HTA bodies* *• Clinical trial comparators should meet both regulatory and HTA requirements* *• What is the HTA equivalent to EMA’s accelerated pathways and conditional approvals?* *• How to align early regulatory and HTA advice procedures?* *• Regulators are relying on the totality of evidence while HTA bodies usually rely only on Head-to-Head evidence*
Different treatment algorithms and national guideline recommendations	*• Clinical Treatment Guidelines differ across Europe* *• Inconsistent definition and relevance of ‘unmet medical need’ within the national HTA procedures*
Different methodological standards for national HTAs	*• Acceptance of Indirect Treatment Comparisons* *• Applied Comparative Treatments* *• Accepted Endpoints* *• Acceptance of pre-defined hierarchical testing* *• Lack of a standardized methodological guidance*
Different national HTA and reimbursement processes	*• Time to market differs across Europe* *• Early Advice and Early Access Programs differ across Europe* *• Acceptance of EU HTA outcomes differs across the various EU countries* *• How to overcome different treatment standards in western* vs *eastern Europe*

There was overlap between some of the free-text comments received in this section of the questionnaire and comments received in the previous section (evolving evidence challenges in oncology HTAs). Therefore, there is some duplication of content within Tables 1 and 2.

### Impact of Europe’s beating Cancer plan on the respondent’s institution

**Fig. 4:**
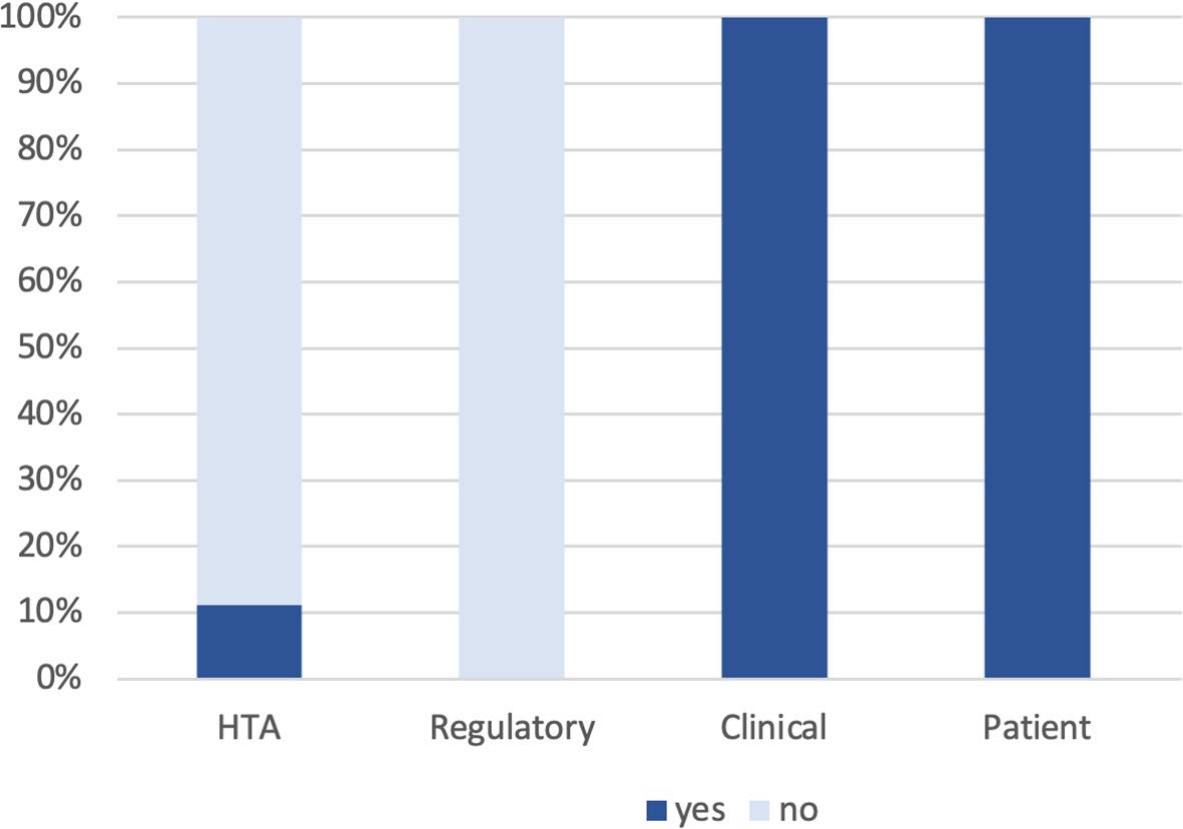
Responses to question: ‘*Have you been involved with any Europe’s Beating Cancer Plan Initiatives?’*. Industry response not shown: Responses suggested that HTA divisions within the industry are not involved in the EBCP but oncology and health policy devisions are involved (personal communication)

**Fig. 5:**
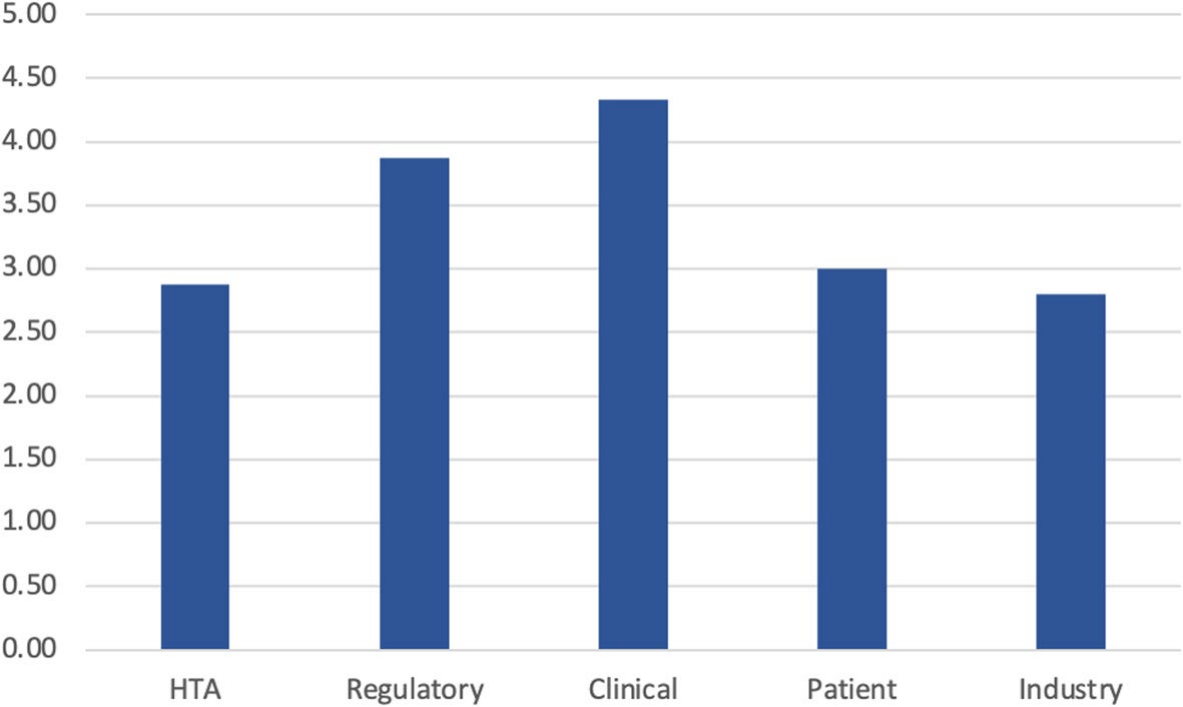
Mean relevance of Europe’s Beating Cancer Plan with regards to each stakeholder group’s activities (scale 1 [low relevance] to 5 [high relevance])

In Figs. 4 and 5 questionnaire responses regarding Europe’s Beating Cancer Plan are presented. While 100% of clinical and patient participants stated that they have been involved to some extent with any national or EU level EBCP initiatives, none of the regulatory agency representatives and industry participants and only one of the HTA bodies replied that they have been involved so far. Also, the perceived relevance of the EBCP differed across the stakeholder groups with clinicians considering it highly relevant (score 4.33) and industry participants considering it less relevant (score 2.80).

Feed-back on the 10 flagship initiatives of the EBCP differed with the *‘Cancer Diagnostic and Treatment for All initiative: access to innovative cancer diagnosis and treatments’* being by far the most frequently mentioned initiative that was considered of high relevance (*n* = 16).

## Discussion

The first chapter within the European Commission’s Pharmaceutical Strategy for Europe [13] is titled: *‘Medicines - a strong ecosystem at an important crossroad’.* With the European regulation on Health Technology Assessment being published in December 2021 [7, 8] and key process milestones such as the establishment of the coordination group and the development of methodological guidelines by the EUnetHTA21 group in progress it is timely to research if and how - at the ‘crossroad of the medicines ecosystem within Europe’ - a joint European HTA may provide an ‘additional benefit’ over the multiplicity of existing national and regional HTAs.

The goal of the EU HTA Regulation is to improve access to life-saving innovative technologies [7]. However, many challenges remain within the implementation of the regulation, which might in fact put this goal at risk. Successful implementation of the new regulation will require involvement from a wide variety of stakeholders including patients. Within our survey, consistency of quantitative and qualitative comments received was high across HTA bodies, regulatory bodies, haematology/oncology societies, patient organisations and industry, indicating that there is a high level of agreement regarding the identified value drivers and evolving evidence challenges in oncology assessments. This finding is not uncommon. In a recent cross-stakeholder web-based survey on data issues in rare disease registries, supporting regulatory decision making also identified well aligned opinions of the various stakeholder groups [14]. However, ongoing integration of all these stakeholder groups within the evolving HTA landscape is critical. Utilizing clinical excellence with the development of high-level and homogeneous European clinical treatment guidelines; patient involvement in the definition of ‘unmet medical need’, ‘patient relevance of endpoints’, and assessment of ‘minimal clinically important difference’; increased collaboration of regulatory and HTA bodies regarding the definition of clinical trial comparators and methodological requirements for endpoints; and close collaboration between evolving European and established national HTA systems were all mentioned as important approaches to overcome the various heterogeneities and inefficiencies of the currently fragmented HTA landscape.

**Fig. 6:**
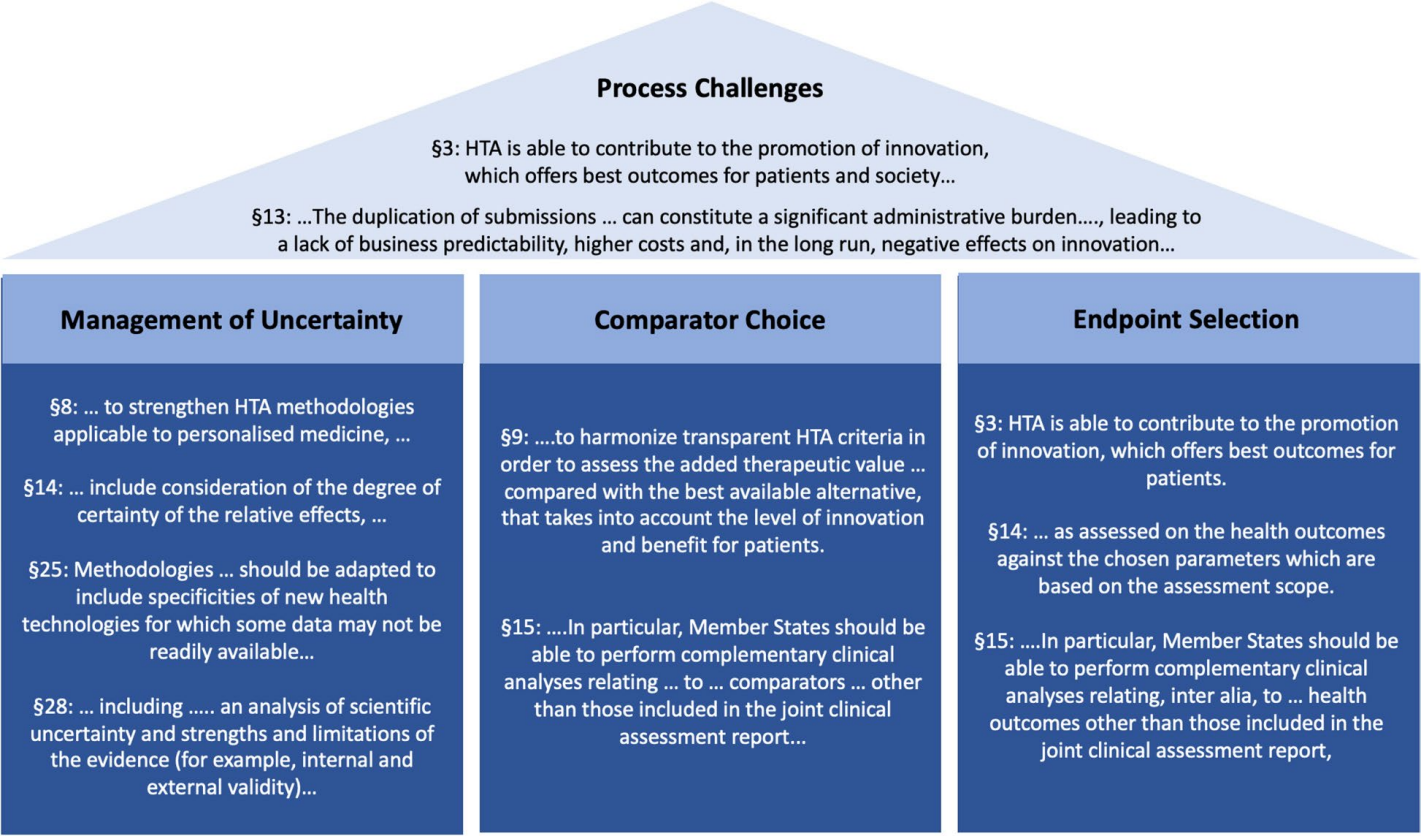
Language derived from the preamble of the EU HTA Regulation regarding the four challenge areas that need to be addressed in order for the EU HTA Regulation to provide an ‘additional benefit’ compared to the status quo of many parallel independent national and subnational assessments

Extensive qualitative responses were received within the section on ‘evolving evidence challenges in oncology HTAs’. Those challenges were grouped into the four categories management of uncertainty, comparator choice, endpoint selection and process challenges, with process challenges impacting all three other methodological challenges. All received comments as well as the four identified priority categories are well aligned with the scope and wording as lined out within the respective paragraphs of the EU HTA Regulation. Figure 6 displays wording derived from the EU Regulation covering the respective challenge categories:

- The recitals of the regulation clearly articulate the need of ***lean and effective processes*** facilitating both a high-quality HTA as well as an increase in predictability and avoidance of duplication and thus fostering innovation within Europe. While the regulation puts very strong emphasis on the delineation between EU level assessment and national responsibility for the HTA appraisal, there is a lack of guidance within the regulation regarding the envisaged EU HTA cooperation framework with the EMA. Within their report on the implementation of the EMA-EUnetHTA work plan 2017–2021 EMA and EUnetHTA provide in-depth insights regarding their discussion of controversial methodological challenges [15]. Continuation of those discussions and clarification of evidence requirements within the regulatory and HTA framework including parallel early advice, early access schemes, as well as regulatory and HTAs is urgently required to ‘ensuring a strong EU voice globally’ in line with chapter 5 of the European Commission’s pharmaceutical strategy for Europe [13, 16, 17].
- The ***management of uncertainty*** after successful EMA approval leading to additional national data requirements as a toll needed for covering the gaps and discrepancies between the regulatory and HTA data needs is another key challenge affecting any future oncology HTA within Europe [18]. While innovative oncology medicines rely on a wealth of genomic data, and treatment algorithms are getting ever more specific, the development of HTA methodologies applicable to personalized medicine, as mentioned in §8 of the preamble of the EU regulation, is still in its infancy [8]. Integration of evidence derived from clinical as well as real-world data sources [19, 20], generating comparative evidence in small patient numbers, and *‘including specificities of new health technologies for which some data may not be readily available’* as outlined in §24 of the preamble of the EU HTA regulation [7, 8] require innovative approaches to the management of uncertainty. Such could be the consideration of new clinical trial designs (e.g., basket trials) [21], the inclusion of data derived from interim analyses in rare e.g., genetic conditions requiring continuous data collection beyond marketing authorization, or the pre-specification of indirect treatment analysis plans.
- Consistency of the applied PICO scheme (Patient/ Intervention/Comparator/Outcomes) and in particular ***comparator selection*** and ***endpoint choice*** were identified in our survey as requiring specific attention in the context of EU HTA. With regards to comparator selection the regulation suggests both a comparison with the best available alternative (preamble §9) as well as the option for member states to deviate and select comparators other than those included in the joint clinical assessment report ( [8], preamble §§9, 15]). Furthermore, several comparators could be included in the EU joint clinical assessment report, of which only a selection might be relevant to a given member state ( [8], preamble §28]. According to EUnetHTA’s ‘Guideline on Comparators and Comparisons’ [22] the comparator treatment applicable across European countries under ideal circumstances would be the reference treatment according to up-to-date high-quality clinical practice guidelines at European or international level. However, the guideline also acknowledges that in many circumstances there is no consensus across European countries, indicating that there are still many challenges to overcome when aiming for consistent and high-level treatment standards across Europe as implied by the European Commission’s vision of a ‘European health union’. Similarly, HTA should enable best outcomes to patients, but member states may include health outcomes other than those included in the joint assessment report. Further, alignment of regulatory and HTA approaches regarding selection of comparator and endpoint selection is not reflected in the regulation but nevertheless of critical importance [23]. Challenges are reaching beyond the mere selection of endpoints, and also include the predefined testing hierarchy that is considered of critical importance e.g., by the French HTA body HAS while not being taken into account at all by the German IQWIG [24, 25].

EUnetHTA21 has devoted distinct project plants to the establishment of a common definition of meaningful clinical endpoints [26] and to comparators and comparisons [27]. Development and alignment of approaches for comparators and (patient-relevant) endpoints with regards to regulatory and HTA requirements was identified as a key challenge within our survey.

Closely linking the two key health initiatives by the European Commission i.e., the EBCP and the HTA Regulation, seems to be an opportunity that has not yet been fully explored. While patients and clinicians are involved in the EBCP, regulatory and HTA bodies’ responses indicated very limited involvement. However, according to the HTA Regulation article 7 cancer medicines and advanced therapy medicinal products are the first health technologies to be subject to joint clinical assessments from 2025 onwards indicating an oncology focus and implying a link to the EBCP [7, 8]. Similarly, one of the core actions within the EBCP is dedicated to ‘ensuring access to high standards in cancer diagnosis and treatment’ with the ‘Cancer Diagnostic and Treatment for All’ initiative already being implemented since 2021 [11]. Despite those obviously overlapping goals between the two European initiatives this apparent lack of cross-fertilization and communication as seen by the responses provided in the questionnaire is striking. Involvement of HTA bodies and regulators in the EBCP activities therefore should be increased to achieve the goal of increased access to cancer diagnosis and treatment for patients, which is common to both initiatives.

While our survey was shared with a wide audience comprising 189 institutions it is a limitation that only 30 responses (16%) were received. A variety of methods such as personalized e-mails, offer of survey results, and two subsequent reminders were leveraged to increase return [28]. However, a 16% return rate was also seen in other unprompted electronic data collections [29]. In addition, responses were received from all five approached stakeholder groups and 17 countries and overall homogeneity of responses across the countries and stakeholder groups was high. Nevertheless, confirmatory research aiming for additional responses in particular from clinical and patient representatives is part of the future research agenda. With the EU HTA regulation being an evolving topic that will gain considerable relevance until 2025 it is also expected the willingness to participate in future research will increase across all involved stakeholder groups.

The outcomes of our semiquantitative survey revealed that while the EU HTA Regulation offers many opportunities there are also challenges that could lead to a risk for patient access to innovation. Achieving ‘additional benefit’ of a joint European HTA will largely depend on the development of a joint and binding methodological framework including homogeneous approaches to key challenge areas such as management of uncertainty, comparator choice, definition of patient-relevant endpoints, as well as on an aligned, transparent and integrated process management that increase predictability and foster innovation carrying an additional value for patients.

## Supplementary Information

**Additional file 1.**

## Data Availability

The datasets used and/or analysed during the current study are available from the corresponding author on reasonable request.
