# Supplementary material for: How can a joint European health technology assessment provide an ‘additional benefit’ over the current standard of national assessments? Insights generated from a multi-stakeholder survey in hematology/oncology
Source: Health Econ Rev. 2022 Jun 2;12:30. doi: 10.1186/s13561-022-00379-7 (PMC9161501; doi:10.1186/s13561-022-00379-7)
Supplement: Supplementary file 1 — Additional file 1. [file 13561_2022_379_MOESM1_ESM.pdf]

Questionnaire - Background Information

1

Which stakeholder group do you represent? *

- ○ Patient
- ○ Clinical
- ○ Regulatory
- ○ Payer
- ○ Industry
- ○ Other

2

What country do you represent? *

3

What is your involvement and / or experience with HTA?

Value Drivers in Oncology Medicines **- 'To Date' -**

4

What are the most relevant drivers of value in oncology medicines (i.e. positive HTA assessments) from your stakeholder point of view? Please rank their respective relevance with numbers 1-5. *

	1 (low relevance)	2	3	4	5 (high relevance)
Clinical Trial Design	◯	◯	◯	◯	◯
Size of comparative effect / magnitude of benefit	◯	◯	◯	◯	◯
Comparator	◯	◯	◯	◯	◯
Right endpoint(s)	◯	◯	◯	◯	◯
Unmet Medical Need	◯	◯	◯	◯	◯
Health Economic Model	◯	◯	◯	◯	◯
Quality of Life (QoL)	◯	◯	◯	◯	◯
Patient Reported Outcomes (PRO)	◯	◯	◯	◯	◯

5

Do you have any specific comments or recommendations regarding the above mentioned value drivers? Please specify:

6

If there are any other value drivers that you consider crucial in oncology medicines (i.e. positive HTA assessments) from your stakeholder point of view, please specify them here:

Evidence Challenges in Oncology Medicines **- 'In Future' -**

7

Based on your understanding of recent diagnostic and therapeutic trends in oncological conditions what would you consider as key challenges for the development of comparative evidence for upcoming oncology medicines? Please rank their respective relevance with numbers 1-5.

A key challenge for future oncology medicines is deriving comparative evidence...
*

	1 (low relevance)	2	3	4	5 (high relevance)
...from small patient numbers e.g., defined by biomarkers	○	○	○	○	○
...from innovative study designs e.g., single-arm, basket or umbrella trials	○	○	○	○	○
...from Real-World Data sources e.g., disease registries	○	○	○	○	○
...from new endpoints e.g., time to next treatment	○	○	○	○	○
...versus a variety of comparative regimens in an increasingly fragmented treatment landscape	○	○	○	○	○

8

Do you have any specific comments or recommendations regarding the above mentioned challenges? Please specify:

9

If there are any other challenges that you consider crucial for deriving comparative evidence for future oncology medicines, please specify them here:

10

What are from your point of view key steps that need to be pursued to address those evidence challenges? *

Heterogeneity of Value Drivers in HTAs on Oncology Medicines **- 'Across Europe' -**

Based on your understanding of the European HTA environment and heterogeneity between national systems, what would you consider as key methodological challenges of HTAs on oncology medicines across Europe that need to be solved within the next three years to further align methodological requirements?

11

Please **specify and provide the rationale** for the methodological heterogeneity across Europe which you consider **most challenging**:
*

12

Please **specify and provide the rationale** for the methodological heterogeneity across Europe which you consider **second most challenging**:

Questions regarding Activities within the **Europe's Beating Cancer Plan**

13

Have you been involved with any national or EU level Europe's Beating Cancer Plan initiatives?

◯ Yes

◯ No

14

If you selected "Yes" in question 13, please provide details on the initiative and your involvement here:

15

How would you rate the overall relevance of the Europe's Beating Cancer Plan with regards to your stakeholder-related activities?

☆ ☆ ☆ ☆ ☆

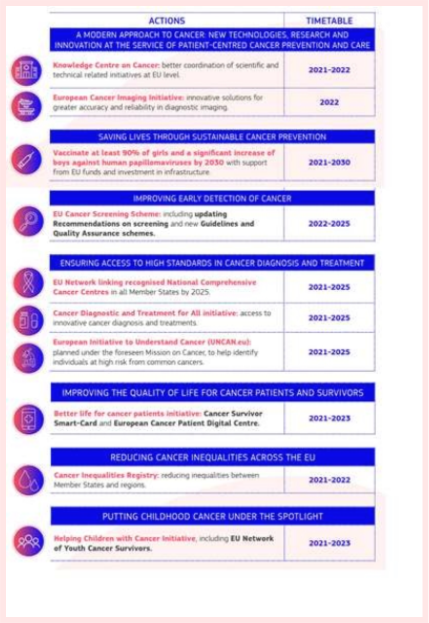

ACTIONS	TIMETABLE
**A MODERN APPROACH TO CANCER: NEW TECHNOLOGIES, RESEARCH AND INNOVATION AT THE SERVICE OF PATIENT-CENTRED CANCER PREVENTION AND CARE**	
**Knowledge Centre on Cancer:** better coordination of scientific and technical related initiatives at EU level.	2021-2022
**European Cancer Imaging Initiative:** innovative solutions for greater accuracy and reliability in diagnostic imaging.	2022
**SAVING LIVES THROUGH SUSTAINABLE CANCER PREVENTION**	
**Vaccinate at least 90% of girls and a significant increase of boys against human papillomaviruses by 2030** with support from EU funds and investment in infrastructure.	2021-2030
**IMPROVING EARLY DETECTION OF CANCER**	
**EU Cancer Screening Scheme:** including **updating Recommendations on screening** and new **Guidelines and Quality Assurance schemes.**	2022-2025
**ENSURING ACCESS TO HIGH STANDARDS IN CANCER DIAGNOSIS AND TREATMENT**	
**EU Network linking recognised National Comprehensive Cancer Centres** in all Member States by 2025.	2021-2025
**Cancer Diagnostic and Treatment for All initiative:** access to innovative cancer diagnosis and treatments.	2021-2025
**European Initiative to Understand Cancer (UNCAN.eu):** planned under the foreseen Mission on Cancer, to help identify individuals at high risk from common cancers.	2021-2025
**IMPROVING THE QUALITY OF LIFE FOR CANCER PATIENTS AND SURVIVORS**	
**Better life for cancer patients initiative: Cancer Survivor Smart-Card** and **European Cancer Patient Digital Centre.**	2021-2023
**REDUCING CANCER INEQUALITIES ACROSS THE EU**	
**Cancer Inequalities Registry:** reducing inequalities between Member States and regions.	2021-2022
**PUTTING CHILDHOOD CANCER UNDER THE SPOTLIGHT**	
**Helping Children with Cancer Initiative,** including **EU Network of Youth Cancer Survivors.**	2021-2023

Within the 10 Flagship activities of the Europe's Beating Cancer Plan, what would you consider the three most relevant activities with regards to the scope of your stakeholder group's work?

(Link to Europe's Beating Cancer Plan Factsheet: https://ec.europa.eu/health/sites/default/files/non_communicable_diseases/docs/cancer-plan_factsheet_en.pdf (https://ec.europa.eu/health/sites/default/files/non_communicable_diseases/docs/cancer-plan_factsheet_en.pdf))
*

17

Are there any components missing in the Europe's Beating Cancer Plan that you would consider highly relevant in oncology? Please specify *

18

How important would you rate Quality of Life (QoL) Assessments in oncology medicines?

☆ ☆ ☆ ☆ ☆

19

Did you / does your organisation work with data derived from digital sources for oncology medicines - e.g., digital QoL questionnaires?

◯ Yes

◯ No

20

What do you expect can be achieved with the Europe's Beating Cancer Plan and EU HTA with regards to patient access to oncology medicines? *

Further Information

If you would like to participate in the inaugural EAA convention or in case we need to clarify any of your responses please fill in your details below.

Your personal information is used solely for the purpose of providing information about the upcoming EAA convention to selected invited participants or in case clarification should be needed for any responses. The personal data will not be included in the analysis of the information collected in the questionnaire. The data collected with this survey will be analysed anonymously, treated confidentially and will not be made available to any third party unless required for the purpose as stated above. All data will be stored in a secured location and will not be stored longer than required.

By participating in this questionnaire, you consent to these terms. Should you wish to withdraw your consent or in order to request the deletion of your personal data please contact the EAA secretariat at *info@r-connect.org (mailto:info@r-connect.org)*.

21

What is your name?

22

What is your email address which we may use in case of questions?

Impressum

This questionnaire about **"Europe's Legislation on Health Technology Assessment & Europe's Beating Cancer Plan - *Bringing the Pieces Together*"** is presented by

The
European Access Academy
e: info@r-connect.org (mailto:info@r-connect.org)
m: +41 76 558 0275
January, 2022

This content is neither created nor endorsed by Microsoft. The data you submit will be sent to the form owner.

Microsoft Forms
